# Role of ectodysplasin signalling in middle ear and nasal pathology in rat and mouse models of hypohidrotic ectodermal dysplasia

**DOI:** 10.1242/dmm.037804

**Published:** 2019-04-25

**Authors:** Jorge del-Pozo, Neil MacIntyre, Ali Azar, Denis Headon, Pascal Schneider, Michael Cheeseman

**Affiliations:** 1 Veterinary Pathology, The Royal (Dick) School of Veterinary Studies, University of Edinburgh, Edinburgh EH25 9RG, UK; 2 Developmental Biology Division, Roslin Institute and The Royal (Dick) School of Veterinary Studies, University of Edinburgh, Edinburgh EH25 9RG, UK; 3 Department of Biochemistry, University of Lausanne, Boveresses 155, CH-1066 Epalinges, Switzerland; 4 Centre for Comparative Pathology & Division of Pathology, University of Edinburgh, Institute of Genetics & Molecular Medicine, Crewe Road, Edinburgh EH4 2XR, UK

**Keywords:** EDAR signalling, *Eda* mouse, *Edaradd* rat, XLHED, Auditory-tube submucosal gland, Otitis media

## Abstract

Patients with mutations in the ectodysplasin receptor signalling pathway genes – the X-linked ligand ectodysplasin-A (*EDA*), the receptor *EDAR* or the receptor adapter *EDARADD* – have hypohidrotic ectodermal dysplasia (HED). In addition to having impaired development of teeth, hair, eccrine sweat glands, and salivary and mammary glands, HED patients have ear, nose and throat disease. The mouse strains *Tabby* (*Eda^Ta^*) and *downless* (*Edar^dl-J/dl-J^*) have rhinitis and otitis media due to loss of submucosal glands in the upper airway. We report that prenatal correction of EDAR signalling in *Eda^Ta^* mice with the agonist anti-EDAR antibody rescues the auditory-tube submucosal glands and prevents otitis media, rhinitis and nasopharyngitis. The sparse- and wavy-haired (*swh*) rat strain carries a mutation in the *Edaradd* gene and has similar cutaneous HED phenotypes to mouse models. We report that auditory-tube submucosal glands are smaller in the homozygous mutant *Edaradd^swh/swh^* than those in unaffected heterozygous *Edaradd^swh/+^* rats, and that this predisposes them to otitis media. Furthermore, the pathogenesis of otitis media in the rat HED model differs from that in mice, as otitis media is the primary pathology, and rhinitis is a later-onset phenotype. These findings in rodent HED models imply that hypomorphic as well as null mutations in EDAR signalling pathway genes may predispose to otitis media in humans. In addition, this work suggests that the recent successful prenatal treatment of X-linked HED (XLHED) in humans may also prevent ear, nose and throat disease, and provides diagnostic criteria that distinguish HED-associated otitis media from chronic otitis media with effusion, which is common in children.

## Abstract (precis)

**Summary:** The *Edaradd^swh/swh^* rat has comparable ear, nose and throat (ENT) pathology to humans with hypohidrotic ectodermal dysplasia, and prenatal correction of EDAR signalling in *Eda^Ta^* mice prevents ENT disease.

## INTRODUCTION

The ectodysplasin signalling pathway comprises a TNF-like ligand [ectodysplasin (EDA)], its transmembrane receptor (EDAR) and an intracellular signal transducer (EDARADD). Loss of signalling due to mutation of genes encoding any component of this linear pathway leads to hypohidrotic ectodermal dysplasia (HED), which is characterised by impaired development of teeth, hair, eccrine sweat glands, and salivary and mammary glands (Kowalczyk-Quintas and Schneider, 2014). The most common form is X-linked HED (XLHED), caused by mutation in the *EDA* gene [Online Mendelian Inheritance in Man (OMIM) number 305100], followed by loss-of-function mutations in *EDAR* (OMIM 604095) or *EDARADD* (OMIM 606603). Rodent models of HED carrying comparable mutations in pathway genes include the mouse strains *Tabby* [*Eda^Ta^*; Mouse Genome Informatics (MGI) number 1856197], *downless* (*Edar^dl-J/dl-J^*; MGI:1856018) and *crinkled* (*Edaradd^cr/cr^*; MGI:1856832), and the sparse and wavy hair (*swh*; *Edaradd^swh/swh^*) rat (Kuramoto et al., 2005, 2011).

Humans with HED have dry nasal passages and concretion of nasal secretions, rhino-sinusitis and nasal infection, otitis media, speech and hearing impairment, and sore throat (Martini et al., 1984; Shin and Hartnick, 2004; Mehta et al., 2007a,b; Callea et al., 2013; Henningsen et al., 2014), which are attributed to impaired development of sinonasal and auditory-tube submucosal glands (SMGs) and reduced ciliary and salivary gland function (Callea et al., 2013). *Eda^Ta^* mice have hypoplastic submandibular salivary glands (Jaskoll et al., 2003), and subsets of nasal/nasopharyngeal SMGs are deleted (Grüneberg, 1971). *Eda^Ta^* and *Edar^dl-J/dl-J^* mice have rhinitis, and otitis media that is associated with deletion of auditory-tube SMGs, leading to reduced auditory-tube gating and ascension of bacteria and foreign body (FB) particles into the bulla. However, the ear, nose and throat pathology in human patients differs from that in *Eda^Ta^* and *Edar^dl-J/dl-J^* mice, in which throat inflammation is a relatively minor feature. In addition, ageing wild-type rats and mice accumulate hyaline droplets in nasal and respiratory and olfactory epithelium, and these are considered to be an incidental finding (Chamanza and Wright, 2015), but in young *Eda^Ta^* and *Edar^dl-J/dl-J^* mice these occur at an unusually high incidence and severity (Azar et al., 2016). This change has no counterpart in human nasal epithelium. Nasal and bulla pathology in *Edaradd^cr/cr^* mice has not been investigated but would presumably phenocopy *Eda^Ta^* and *Edar^dl-J/dl-J^* mice.

The heterozygous *Edaradd^swh/+^* rat has a wild-type appearance, but the homozygous *Edaradd^swh/swh^* rat shares most cutaneous and dental phenotypes with HED mutant mouse strains. However, HED mice have a hairless tail with a terminal tail kink, whereas *Edaradd^swh/swh^* rats have a haired tail, a low penetrance of a terminal tail kink and lack a bald patch behind the ear (Kuramoto et al., 2005, 2011). Nasal and bulla pathology have not been investigated in *Edaradd^swh/swh^* rats, but differences between the nasal histology and anatomy in mice and rats (Chamanza and Wright, 2015), as well as rats having larger nasal passages, nasopharynx and bullae, could conceivably alter predisposition to disease in the *Edaradd^swh/swh^* rat model.

Prenatal administration of agonist anti-EDAR antibodies (mAbEDAR1) to *Eda^Ta^* mice rescues cutaneous and dental HED phenotypes (Kowalczyk et al., 2011). The mouse auditory-gland SMGs form at embryonic day (E)18-E19 (Grüneberg, 1971; Park and Lim, 1992). The *Edar* gene is expressed in the auditory-tube SMGs in postnatal day (P)21 *Edar*^Tg951/951^ mice (Azar et al., 2016), which have high levels of *Edar* expression (Majumder et al., 1998; Mou et al., 2008; Chang et al., 2009). On the assumption that the *Edar* gene is expressed in gland primordia, we hypothesized that prenatal correction of EDAR signalling would also rescue auditory-tube SMGs. Recently, human XLHED phenotypes (deficient development of sweat glands and sweating function, Meibomian glands and some tooth germ buds) have been corrected by prenatal treatment of the fetus via intra-amniotic injection with a recombinant protein that includes the receptor-binding domain of EDA (Schneider et al., 2018).

This work focuses on two related aims: (1) to test whether agonist anti-EDAR antibodies could rescue auditory-tube SMGs in *Eda^Ta^* mice and thereby prevent otitis media; and (2) to explore the comparative nasal and bulla pathology in the *Edaradd^swh/swh^* rat.

## RESULTS

### Otitis media, hyaline droplet change, rhinitis and nasopharyngitis in *Eda^Ta^* mice

**Fig. 1.**
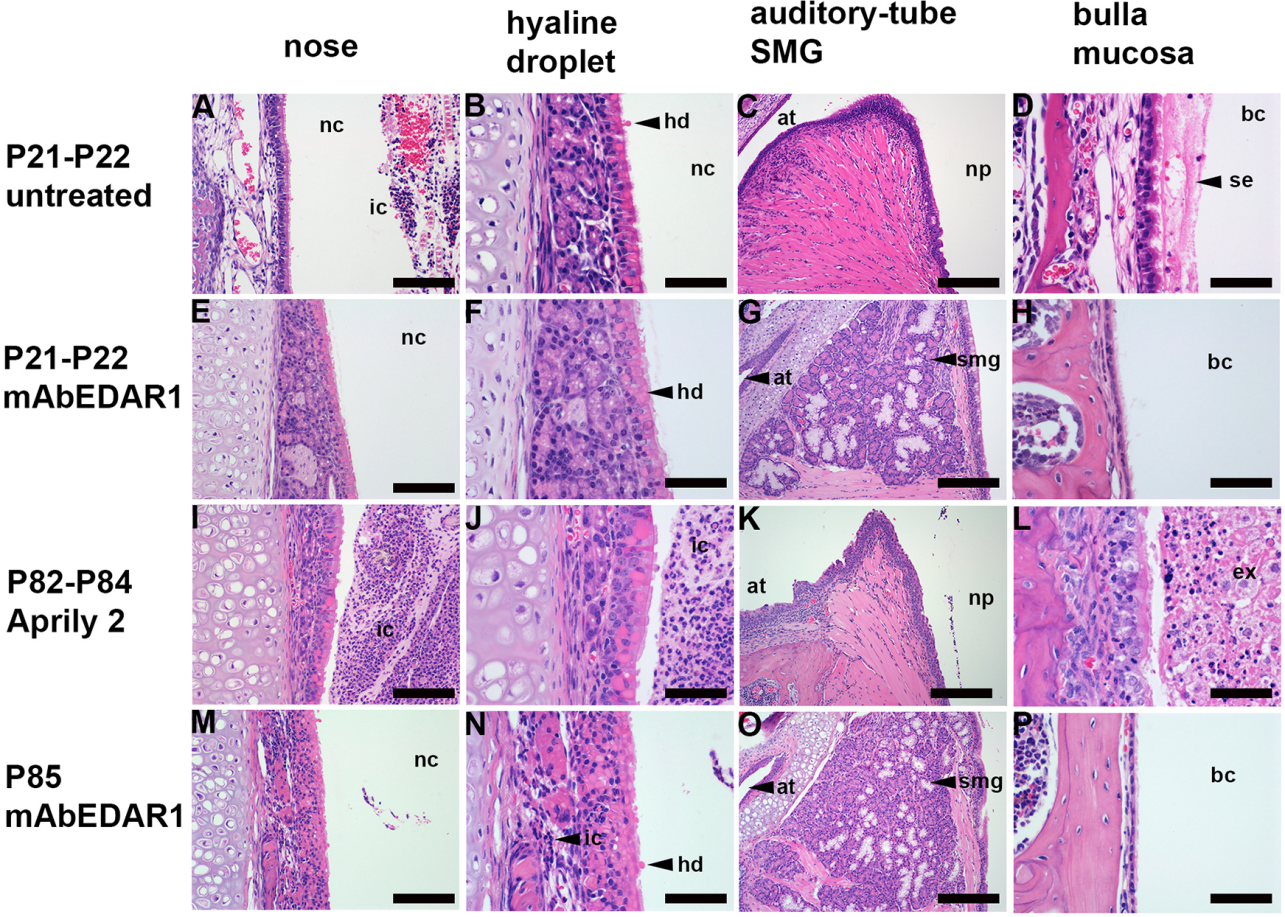
**Prenatal treatment with agonist anti-EDAR antibody rescues auditory-tube submucosal glands (SMGs) and otitis media in *Eda^Ta^* mice.** (A-D) Untreated P21 *Eda^Ta^* mice. (E-H) P21-P22 *Eda^Ta^* mice treated with agonist anti-EDAR antibody (mAbEDAR1). (I-L) P82-P84 *Eda^Ta^* mice treated with isotype control Aprily-2 (mouse IgG1 anti-hAPRIL). (M-P) P85 *Eda^Ta^* treated with mAbEDAR1. Rhinitis (A,I,J) is characterised by heavy inflammatory cell exudation into the nasal cavity and is prevented (E,F) by mAbEDAR1 treatment except in a single P85-treated mouse shown (M,N), in which there is light infiltration of mucosa with inflammatory cells (shown in N). (B,F,J,N) Hyaline droplet change in nasal respiratory epithelium occurs in untreated, mAbEDAR1 and Aprily-2-treated mice. mAbEDAR1 rescues auditory-tube SMGs (G,O) and prevents otitis media (H,P). Otitis media is characterised by serous effusion in untreated P21 *Eda^Ta^* mice (D) and suppurative exudation in Aprily-2-treated P85 *Eda^Ta^* mice (L), whereas (H,P) P21 and P85 *Eda^Ta^* mAbEDAR1-treated mice have healthy air-filled bulla cavity lined by slender mucosa. Panels E and F, I and J, K and L, and M and N are low- and high-magnification image pairs. at, auditory tube; bc, bulla cavity; ex, exudate; hd, hyaline droplets; ic, inflammatory cells; nc, nasal cavity; np, nasopharynx; se, serous effusion; smg, submucosal gland. Scale bars: (C,G,K,O) 200 µm; (A,E,I,M) 100 µm; (B,D,F,H,J,L,N,P) 50 µm.

**Fig. 2.**
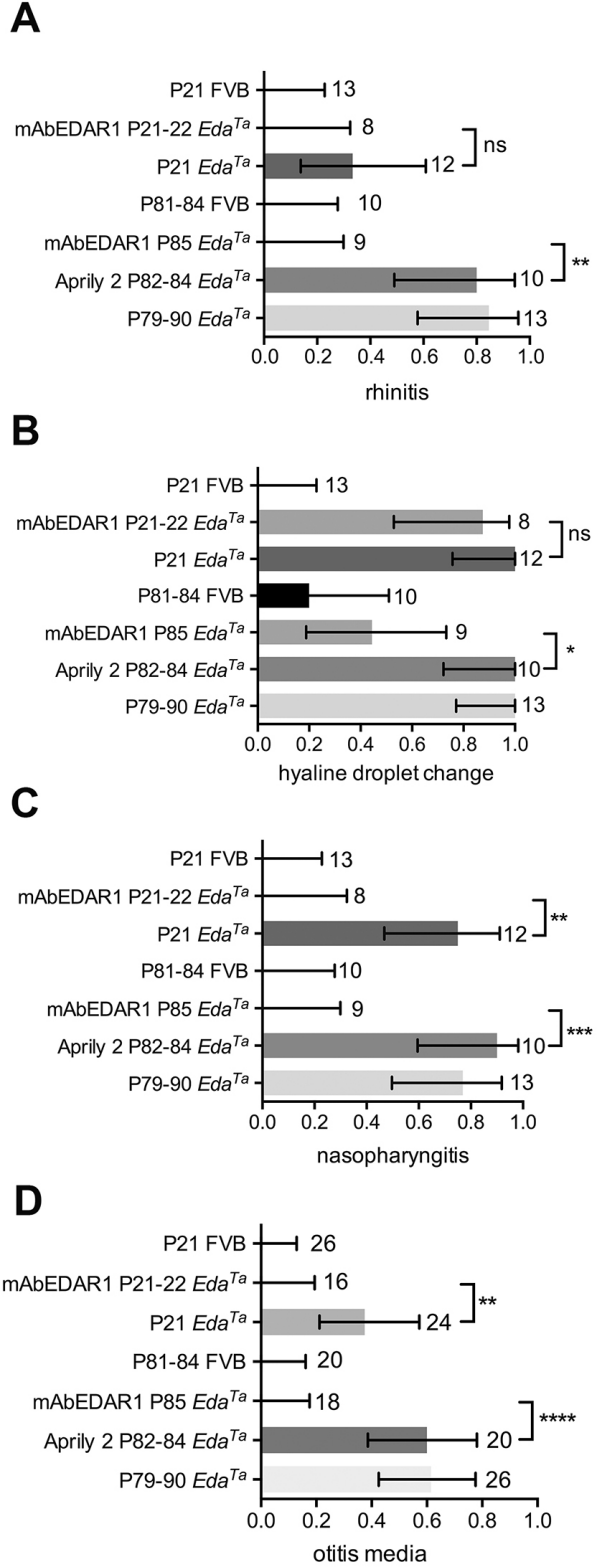
**Prevalence of nasal, nasopharynx and bulla pathology in *Eda^Ta^* mice.** Proportions of FVB and *Eda^Ta^* mice with (A) rhinitis, (B) hyaline droplet change in nasal respiratory epithelium, (C) nasopharyngitis and (D) otitis media. The prevalence of rhinitis, nasopharyngitis and otitis media is significantly reduced by treatment with mAbEDAR1 in (*n*=2 prenatal only, and *n*=6 pre- and postnatally treated) P21-P22 *Eda^Ta^* mice and in P85 *Eda^Ta^* mice, compared to untreated and Aprily-2-antibody-treated controls. mAbEDAR1 treatment reduced the prevalence of hyaline droplet change at P85. FVB mice had no rhinitis, nasopharyngitis or otitis media, and hyaline droplet change was only evident in a small proportion of FVB mice at P81-P84. The numbers in the body of the graphs represent the number of tissues examined (nasal, nasopharynx and bullae) in each cohort. The histogram bar represents the mean proportion, and error bars the 95% confidence interval for each proportion. Frequency data were analysed with Fisher's exact tests. Two-tailed tests; ns, not significant (*P*>0.05), *P<0.05, **P<0.01, ***P<0.001, ****P<0.0001.

The frequency of rhinitis, nasopharyngitis and otitis media in *Eda^Ta^* mice at weaning age is <40% and rises to >80% at 7-17 weeks of age; hyaline droplet accumulation in nasal respiratory epithelium is fully penetrant at weaning age (Azar et al., 2016). To standardize experimental observations, we therefore chose an early time point of P21-P22 and a late time point of P79-P90, when pathology approaches maximal penetrance. Initially, we examined untreated *Eda^Ta^* mice and the spontaneous background pathology in FVB mice (the background inbred genetic line for the *Eda^Ta^* strain). Untreated P21 (*n*=12) and P79-P90 (*n*=13) *Eda^Ta^* mice had typical features of HED characterised by sparse hair coat and a hairless tail with a terminal kink. Auditory-tube SMGs were deleted in all P21 and P79-P90 *Eda^Ta^* mice, and the proportion of bullae with histological otitis media was 0.38 (9/24) at P21 and 0.61 (16/26) at P79-P90. The bulla cavity contained serous bulla effusion at P21 (Fig. 1). The proportion of P21 and P79-P90 *Eda^Ta^* mice with nasal inflammation (rhinitis) was 0.33 (4/12) and 0.85 (11/13), and inflammation of the nasopharynx (nasopharyngitis) 0.75 (9/12) and 0.77 (10/13), respectively. All *Eda^Ta^* mice had hyaline droplet accumulation in nasal respiratory epthelium. FVB (*n*=13 P21 and *n*=10 P81-P84) control mice had normal wild-type hair coats and tails, histologically normal cutaneous and auditory-tube SMGs, and nasal, nasopharynx and bulla pathology was absent, whereas hyaline droplet change was evident in a low proportion (0.2) at P81-P84 (Fig. 2).

### Otitis media is rescued in *Eda^Ta^* mice by agonist anti-EDAR antibodies

#### Prenatal treatment

In keeping with Grüneberg (1971) and Park and Lim (1992), we found gland primordia in E18.5 wild-type mice. Primordia appear as tissue buds that extend from the nasopharynx and auditory-tube epithelium into submucosal connective tissue. E18.5 gland primordia show high expression of the *Edar* gene by *in situ* hybridization (ISH), but relatively low expression of *Foxj1* (a ciliated cell marker) and *Bpifa1* (an antibacterial/surfactant product of the adult murine SMG acini; Bartlett et al., 2015). P1 gland primordia stain by immunohistochemistry (IHC) for keratin 5 (K5), K8 and K19, and have a laminin-positive basal lamina (Fig. S1). K5-positive basal cells are a putative stem cell phenotype; K8 is a primary keratin and K19 is a secondary keratin of simple epithelial cells.

In agreement with Kowalczyk et al. (2011), we found that prenatal administration of mAbEDAR1 to pregnant *Eda^Ta^* females at E10.5 and E17.5 rescued the major macroscopic cutaneous HED phenotypes in 11 (*n*=2 P22 and *n*=9 P85) offspring mice; a minor tail kink was observed in one treated *Eda^Ta^* pup but the remainder had wild-type cutaneous features. In addition, we found that auditory-tube SMGs were rescued by prenatal treatment in all (*n*=2) P22 and (*n*=9) P85 mice. Otitis media and nasopharyngitis were absent in all treated *Eda^Ta^* mice. One of nine mice at P85 had mild rhinitis (Fig. 1). Hyaline droplet change was present in 2/2 P22 and 5/9 P85 treated *Eda^Ta^* mice (Fig. 2).

#### Pre- and postnatal treatment

Nasal respiratory mucosa hyaline droplet accumulation is a postnatal phenotype. It was absent in P5 (*n*=1) and P6 (*n*=2) *Eda^Ta^* mice but fully penetrant in untreated P21-P22 *Eda^Ta^* mice. We therefore tested a pre- and postnatal mAbEDAR1 dosage regimen by treating six pups at P1, P7 and P14 (in addition to E10.5 and E17.5) with the specific aim of seeing whether this could also rescue hyaline droplet change. Cutaneous HED phenotypes were rescued in all but one of six P21 *Eda^Ta^* pups that had a sparse hair coat. As expected from the previous experiment, auditory-tube SMGs, rhinitis and nasopharyngitis were rescued in 6/6 mice; none had otitis media. However, hyaline droplet change remained fully penetrant and this raised the question of whether nasal respiratory epithelium and medial nasal glands were capable of responding to EDAR signalling during the postnatal period. We tested this by showing *Edar* ISH signals at P5 (Fig. S1).

### Isotype control antibodies fail to rescue upper respiratory tract phenotypes in *Eda^Ta^* mice

Isotype control antibody (Aprily-2) was administered to two pregnant *Eda^Ta^* females and all (*n*=10) offspring had typical cutaneous, upper airway and bulla HED phenotypes at P82-P84. The affected proportions were 0.8, 1, 0.9 and 0.60 for rhinitis, nasal hyaline droplet change, nasopharyngitis and otitis media, respectively, and otitis media was characterised by suppurative exudation (Figs 1,2). We also confirmed the presence of Meibomian glands, footpad eccrine sweat glands and tail hair follicles with sebaceous glands in the mAbEDAR1 treatment groups, and their absence in Aprily-2-treated *Eda^Ta^* mice (Fig. S2).

### Otitis media in *Edaradd^swh/swh^* rats is associated with smaller auditory-tube SMGs

**Fig. 3.**
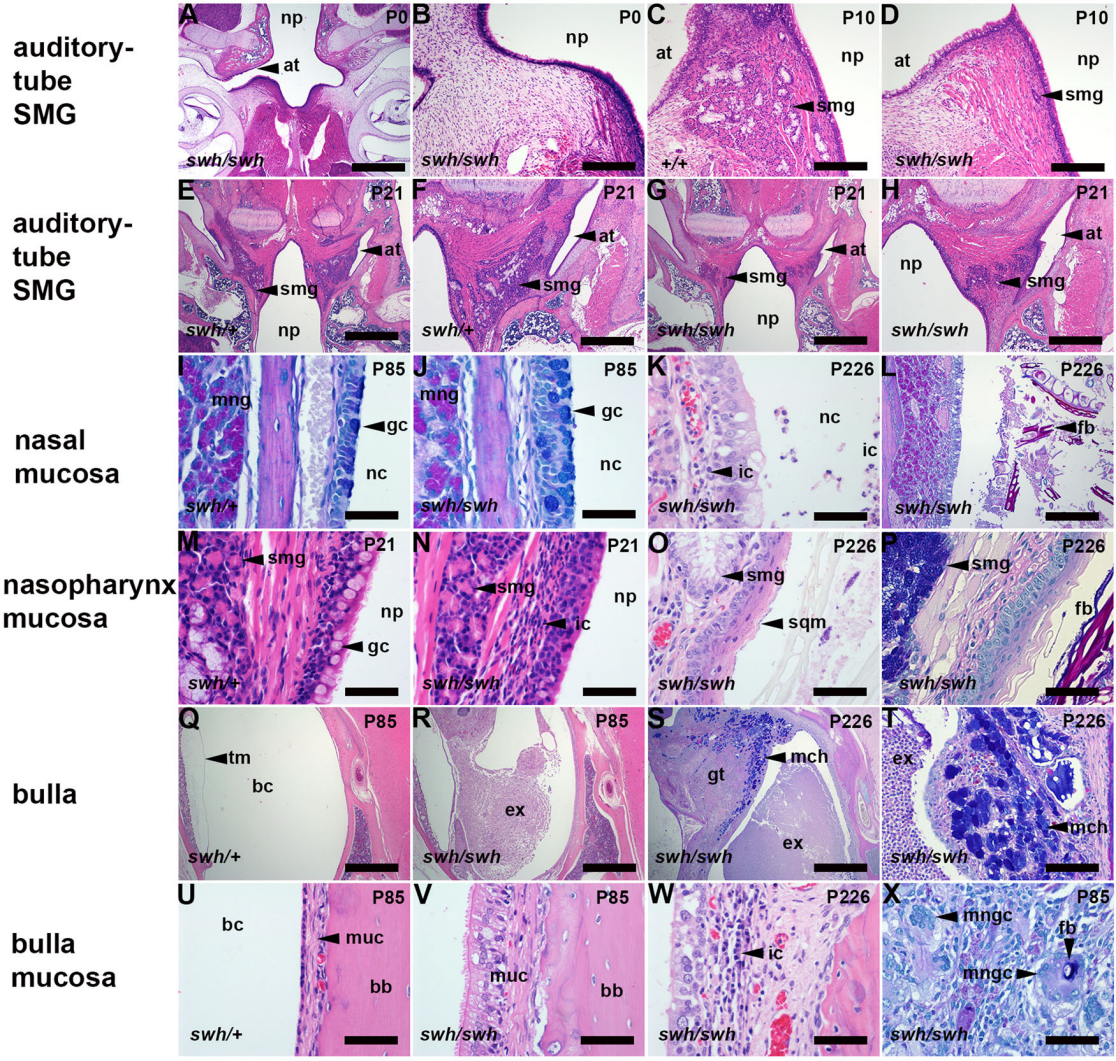
**Auditory-tube submucosal gland (SMG) changes in *Edaradd^swh/swh^* rats are associated with nasal, nasopharynx and bulla pathology.** (A,B) Auditory-tube SMGs are not apparent in a P0 *Edaradd^swh/swh^* rat, and reduced in size at P10 (C,D) and P21 (E-H) compared to *Edaradd^swh/+^* rats. (I,J) Nasal respiratory epithelium is similar in P85 *Edaradd^swh/+^* and *Edaradd^swh/swh^* rats, with numerous goblet cells (AB-PAS stain), and rhinitis and hyaline droplet change are absent. (K,L) P226 *Edaradd^swh/swh^* rat with rhinitis associated with (K) mucosal inflammation and (L) FB particles and exudation into the nasal passages (AB-PAS stain). (M) The nasopharynx of the P21 *Edaradd^swh/+^* rat is lined with goblet cells but in (N) P21 *Edaradd^swh/swh^*, epithelial goblet cells are lost and there is an inflammatory cell infiltration in submucosal connective tissue. (O) P226 *Edaradd^swh/swh^* nasopharynx with epithelial squamous metaplasia and (P) intraluminal FB particles (AB-PAS stain). (Q) Healthy air-filled bulla cavity in a P85 *Edaradd^swh/+^* rat, and otitis media in *Edaradd^swh/swh^* bullae at P85 (R) and P226 (S) characterized by intraluminal exudation and inflamed granulation tissue and mucous cell hyperplasia (AB-PAS stain). (T) Higher-power magnification of mucous cell hyperplasia (AB-PAS stain). (U) Normal slender mucosa in a P85 *Edaradd^swh/+^* bulla. (V) P85 *Edaradd^swh/swh^* bulla mucosa with ciliated-cell hypertrophy and thickened mucosa. (W) P226 *Edaradd^swh/swh^* bulla mucosa with lymphocyte and plasma cell infiltrates. (X) FB and multinucleate giant cell reaction in granulation tissue in a P85 *Edaradd^swh/swh^* bulla (AB-PAS stain). at, auditory tube; bb, bulla bone; bc, bulla cavity; ex, exudate; fb, foreign body; gc, goblet cell; gt, granulation tissue; ic, inflammatory cells; mch, mucous cell hyperplasia; mng, medial nasal gland; mngc, multinucleate giant cell; muc, mucosa; nc, nasal cavity; np, nasopharynx; sqm, squamous metaplasia; smg, submucosal gland; tm, tympanic membrane. Scale bars: (A,E,G,Q-S) 1000 µm; (F,H) 500 µm; (B-D,L) 200 µm; (X) 100 µm; (I-K,M-P,T-W) 50 µm.

**Fig. 4.**
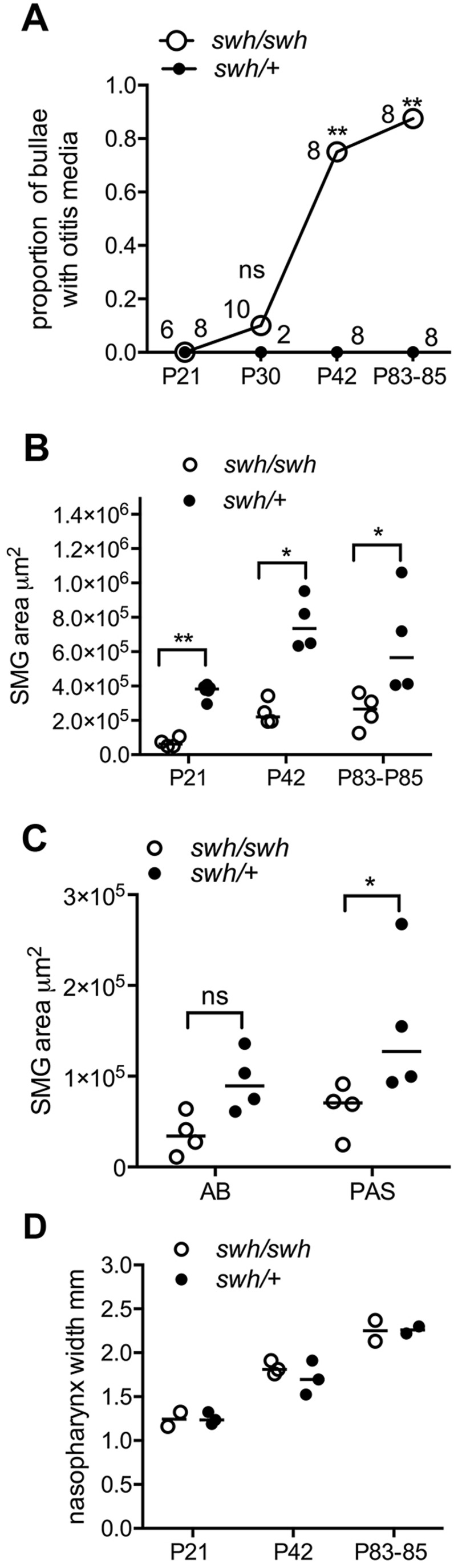
**Otitis media in *Edaradd^swh/swh^* rats is associated with smaller auditory-tube submucosal glands (SMGs).** (A) The prevalence of otitis media in *Edaradd^swh/swh^* rats is indicated by the proportion of affected bullae and increases significantly from P30 to P42 and P83-P85 compared with age-matched *Edaradd^swh^^/^^+^* littermates. Data are represented as points. The number to the left of each data point indicates the number of *Edaradd^swh/swh^* bullae examined, and those to the right are for *Edaradd^swh/+^* bullae. Frequency data were analysed using Fisher's exact tests. (B) Auditory-tube SMGs were smaller in *Edaradd^swh/swh^* than *Edaradd^swh/+^* rats at the P21, P42 and P83-P85 time points. Two or three rats of each genotype were analysed at each time point. The area of each gland was measured in histological step sections and the right and left glands were measured separately. (C) In P83-P85 *Edaradd^swh/swh^* auditory-tube SMGs, the area occupied by PAS-positive acini is reduced, but this trend does not achieve statistical significance for AB-positive mucus. (D) Nasopharynx width increases with age, but median width is comparable between *Edaradd^swh/swh^* and *Edaradd^swh/+^* for each time point. Data in graphs B-D are represented as points and the bar the median. Data in B and C were analysed with Mann–Whitney tests. Two-tailed tests were used throughout. ns, not significant (*P*>0.05); *P<0.05, **P<0.01.

Auditory-tube SMGs were present in *Edaradd^swh/swh^* rats (*n*=21), but were not apparent in serial sections of one P0 pup. In a qualitative comparison between two P10 littermates, the SMGs appeared smaller in the *Edaradd^swh/swh^* pup than those in the *Edaradd^+/+^* littermate (Fig. 3). The auditory-tube SMGs appear as nests and sheets of cells separated by bands of striated muscle. The glandular epithelium includes serous acini and Alcian Blue (AB)-positive mucous cells. Serous cells have periodic acid schiff (PAS)-positive cytoplasmic granules that are also positive for the antibacterial/surfactant protein Bpifa1 (Fig. S3). Auditory-tube SMGs in P21, P42 and P83-P85 *Edaradd^swh/swh^* rats had smaller area profiles compared to those in aged-matched *Edaradd^swh/+^* littermates (Figs 3,4). P83-P85 *Edaradd^swh/swh^* auditory-tube SMGs had a smaller area of PAS-positive acini but the reduction in AB-positive mucus did not achieve statistical significance (Figs 3,4, Fig. S3). Nasopharynx widths were comparable between genotypes and were ∼1.2 mm, ∼1.9 mm and ∼2.4 mm at P21, P42 and P83-P85, respectively, in aged-matched littermates (Fig. 4).

Up until P21, *Edaradd^swh/swh^* bullae were healthy (*n*=2 P0, *n*=2 P10, *n*=2 P13, *n*=6 P21) but otitis media was observed in 1/10, 6/8, 7/8 and 4/4 bullae at P30, P42, P83-P85 and P226-P250, respectively. Littermate controls had normal healthy bullae (*n*=8 P21, *n*=2 P30, *n*=8 P42, *n*=8 P83-P85 *Edaradd^swh/+^*; *n*=4 P83-P84 *Edaradd^+/+^*) (Fig. 4). Otitis media in >P42 *Edaradd^swh/swh^* rats is characterised by suppurative exudation into the bulla cavity, inflammatory thickening of the bulla mucosa, granulation tissue formation, macrophage and multinucleate giant cell reaction to FB particles, and, in two (P226 and P250) *Edaradd^swh/swh^* rats, marked mucous cell hyperplasia (Fig. 3).

Unlike *Eda^Ta^* mice, the nasal respiratory epithelium in *Edaradd^swh/swh^* rats did not show hyaline droplet change, and rhinitis was absent or minimal in animals up to P83-P85. P21, P30, P42 and P83-P85 *Edaradd^swh/swh^* rats had neutrophil infiltration of nasopharynx mucosa. Two (P226 and P250) *Edaradd^swh/swh^* females had marked rhinitis, nasopharyngitis with squamous metaplasia; one of these also had a heavy load of FB particles in the nasopharynx lumen (Fig. 3), and both had FB particles in the auditory-tube lumen.

### *Edaradd* expression in rat tissues and HED phenotypes in *Edaradd ^swh/swh^* rats

**Fig. 5.**
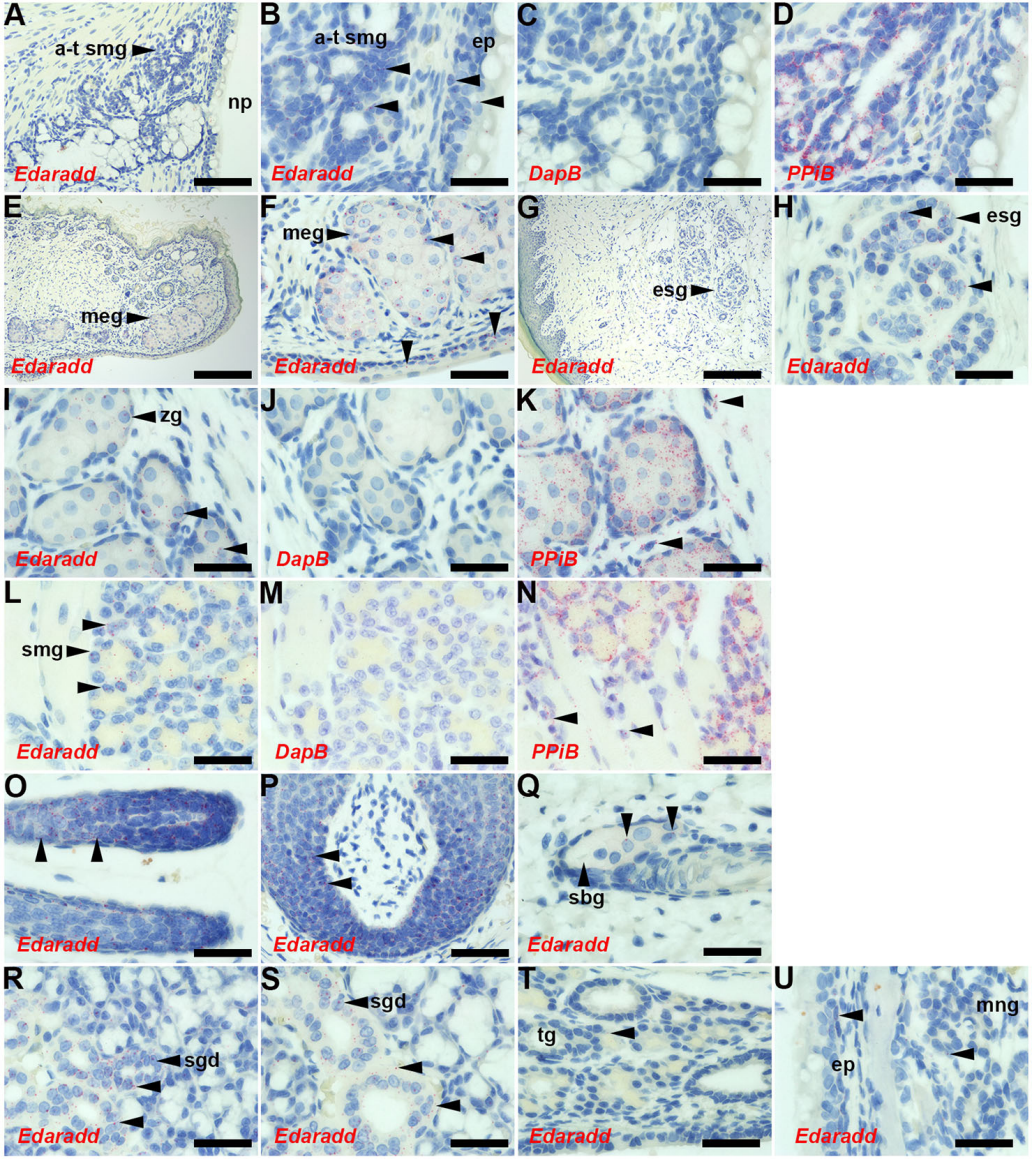
**Expression of *Edaradd* in P10 and P13 rat tissues.** (A-D,I-K,U) P10 *Edaradd^+/+^* and (E-H,L-T) P13 *Edaradd^swh/+^* tissues. ISH signals appear as red dots (unlabelled arrowheads in B,F,H,I,K,L,N-U), and the ISH probe is indicated in each panel. *Edaradd* expression is restricted to epithelia and glands; no signals are given with the negative control probe *DapB*. The positive control probe, *PPiB*, gives strong signals in connective tissue cell populations as well as epithelial tissues (arrowheads in K,N). *Edaradd* ISH signals (arrowheads) are seen in (B) auditory-tube SMG and epithelium (B-D are higher-power images of A); (E,F) Meibomian gland and conjunctiva epithelium; (G,H) footpad with eccrine sweat gland; (I-K) Zymbal's gland; (L-N) tongue SMG; (O) anagen hair follicle; (P) whisker hair follicle; and duct epithelium in (R) sublingual and (S) submandibular salivary glands. *Edaradd* ISH signals in (Q) sebaceous gland, (T) tracheal gland and (U) nasal respiratory epithelium and medial nasal gland are low. a-t smg, auditory-tube SMG; ep, epithelium; esg, eccrine sweat gland; meg, Meibomian gland; mng, medial nasal gland; np, nasopharynx; sbg, sebaceous gland; sgd, salivary gland duct; tg, tracheal gland; zg, Zymbal's gland. Scale bars: (G) 200 µm; (A,E) 100 µm; (P) 50 µm; (B-D,F-O,Q-U) 20 µm.

*Edaradd* ISH signals were detected in P10 *Edaradd^+/+^* auditory-tube SMGs, nasopharyngeal epithelium and the Zymbal's gland associated with the outer ear canal, but expression in nasal respiratory epithelium and medial nasal glands was low (Fig. 5). The histological features of HED in *Edaradd^swh/swh^* rats were essentially those described by Kuramoto et al. (2005, 2011). However, we found hypoplastic foci of eccrine sweat glands in some footpad sections, and some tongue sections had serous SMGs (Fig. S4). The *Edaradd* gene was expressed in these tissues in P13 *Edaradd^swh/+^* rats, as well as in Meibomian glands and conjunctiva epithelium, the ductal epithelium in submandibular and sublingual salivary glands, anagen hair follicles, and whisker follicles, but expression was weak in sebaceous glands and tracheal glands (Fig. 5).

## DISCUSSION

We report that prenatal administration of agonist anti-EDAR antibody to *Eda^Ta^* mice rescues rhinitis, nasopharyngitis, auditory-tube SMGs and otitis media. These results provide further evidence for the importance of EDAR signalling in murine upper respiratory tract SMG development, and the role of these glands in the protection of nasal cavity, nasopharynx and bullae (Azar et al., 2016). We also demonstrate the first non-vaccine intervention to prevent otitis media in an animal model. Auditory-tube SMG primordia arise at E18-E19 (Grüneberg, 1971; Park and Lim, 1992) but are missing in *Eda^Ta^* mice (Grüneberg, 1971). We show that the *Edar* gene is expressed in wild-type E18.5 gland primordia. We infer that prenatal (E10.5 and E17.5) administration of agonist anti-EDAR antibody acts via the receptor EDAR to initiate gland primordia growth in *Eda^Ta^* mice. Prenatal rescue of auditory-tube SMGs provides long-term bulla protection, indicating that continuous EDAR signalling is not required for adult gland function. Auditory-tube SMGs are present in newborn children (Orita et al., 2002; Berger, 1993), implying that novel prenatal correction of XLHED phenotypes (Schneider et al., 2018) may also aid upper respiratory tract SMG development, thereby reducing predisposition to chronic nasal and bulla pathology.

Hyaline droplets arise in *Eda^Ta^* nasal respiratory epithelium between P5/P6 and P21/P22 but are only evident at a low level in >P80 FVB mice. *Edar* is expressed in P5 *Eda^Ta^* nasal respiratory epithelium and medial nasal glands, albeit at lower levels than embryonic auditory-tube gland primordia, suggesting that nasal epithelia are potentially responsive to EDAR signalling. However (pre- and) postnatal (P1, P7 and P14) administration of agonist anti-EDAR antibody does not rescue hyaline droplet change at P21, but there appears to be a partial rescue of hyaline droplet change at P85. This may represent an effect of EDAR signalling on basal cells that give rise to mature ciliated respiratory epithelium cells in normal cell turnover. Agonist anti-EDAR antibody treatment reduces rhinitis more than hyaline droplet accumulation, suggesting that other factors may work in concert with nasal inflammation to cause this cellular change. Hyaline droplet change has been described in ageing rats (St. Clair and Morgan, 1992), but we did not observe it in *Edaradd^swh/swh^* rats with otitis media. Although the aetiology of early hyaline droplet change in HED mice remains unexplained, it does not appear to be central to nasal and bulla inflammatory disease in rodent models.

The *Edaradd* gene is expressed in auditory-tube SMGs of P10 *Edaradd^+/+^* rats. *Edaradd^swh/swh^* auditory-tube SMGs were not apparent at birth (in the single pup examined) and were subsequently smaller than those of *Edaradd^swh/+^* and *Edaradd^+/+^* littermates. A detailed embryonic study is required to establish the normal time course of auditory-tube SMG development, but the available data suggests that *Edaradd^swh/swh^* glands have postnatal growth retardation. Eccrine sweat gland and tongue salivary SMGs express the *Edaradd* gene and are also hypoplastic in *Edaradd^swh/swh^* rats. This may be interpreted as support for the suggestion that *swh/swh* mutation is a loss-of-function rather than a null mutation (Kuramoto et al., 2011). In particular, *Edaradd^swh/swh^* rats, in contrast to *Eda^Ta^* mice, still have a hairy tail. Blocking endogenous EDA protein in developing wild-type mice with an EDA-blocking antibody induced a full-blown HED phenotype, with the exception of the tail, which remained hairy (Kowalczyk-Quintas et al., 2014). Residual EDAR signalling in anti-EDA-treated mice, or in untreated *Edaradd^swh/swh^* rats, may be sufficient to support tail hair genesis. Indeed, EDAR stimulation in newborn *Eda^Ta^* mice for as short as 3.5 h was sufficient to induce tail hair (Swee et al., 2009). As expected, *Edaradd* gene expression maps to many of the epithelia, appendages and glands that are affected in HED.

Otitis media was not observed in *Edaradd^swh/+^* and *Edaradd^+/+^* rats but was first observed at P30 in *Edaradd^swh/swh^* rats, and its prevalence increased significantly by P42. The auditory-tube SMGs in *Edaradd^swh/swh^* rats are smaller than those in littermate control *Edaradd^swh/+^* rats in this critical period. Affected bullae contain pro-inflammatory FB particles that act as fomites. The smaller auditory-tube SMGs in P83-P85 *Edaradd^swh/swh^* rats have significantly reduced profiles of PAS-positive acinar secretory granules, which may translate to reduced secretion of proteins such as antibacterial/surfactant protein Bpifa1 (Musa et al., 2012), which, in common with the mouse, is a product of the adult auditory-tube SMGs (Bartlett, et al., 2015). Such deficits potentially contribute to impaired auditory-tube gating function and bulla defence. The rat model indicates that functional deficiency, as well as deletion, of auditory-tube SMGs can predispose to otitis media. This makes it likely that hypomorphic as well as null mutations in EDAR signalling pathway genes in humans will impact on gland structure and function, and predispose individuals to otitis media.

The pathogenesis of HED-associated otitis media differs in rat and mouse models. Otitis media in *Edaradd^swh/swh^* rats was established by P42 but rhinitis and impaired nasal mucociliary clearance, marked by FB-particle accumulation in the nasopharynx, was only observed in >P200 *Edaradd^swh/swh^* rats. In contrast, otitis media, rhinitis and impaired mucociliary clearance are evident in P21 *Eda^Ta^* and *Edar^dl-J/dl-J^* mice (Azar et al., 2016). Although *Edaradd^swh/swh^* rats have partial auditory-tube SMG function, the bullae are exposed to the ascension of FB particles. Better nasal/nasopharynx mucociliary clearance in rats may be conferred by a high density of goblet cells in the nasal septum (Chamanza and Wright, 2015) and a larger (wider) nasopharynx, up to ∼2.4 mm in P83-P85 rats compared to ∼1.2 mm in mature 10-week-old C57BL/6J mice (Bab et al., 2007). However, larger auditory tubes may require higher levels of SMG protection. Morphometric analysis of auditory-tube sections is of limited value in assessing auditory-tube function because the tube is normally closed and its opening and closing is achieved by muscle action. Formalin fixation can cause artefactual muscle contracture, potentially distorting the *in vivo* contours of the tube.

In conclusion, we report that the agonist anti-EDAR antibody rescues auditory-tube glands in *Eda^Ta^* mice and prevents rhinitis and otitis media. There are broad similarities in HED pathology in rat and mouse models; however, otitis media in rats is the primary pathology and is associated with auditory-tube SMG deficiency. Certain histological features in rats, such as mucous cell hyperplasia in chronic otitis media, show a closer resemblance to humans and underline the value of using diverse species models in otitis media research (Li et al., 2013). The finding of HED-associated otitis media in rats as well as mice suggests that dogs and cattle with HED (Hadji Rasouliha et al., 2018; Kowalczyk et al., 2011; Waluk et al., 2016; Karlskov-Mortensen et al., 2011; Seeliger et al., 2005) may also have otitis media. A better understanding of interspecies differences in HED-associated otitis media will increase the confidence in translating findings to human patients, in which the direct study of auditory-tube SMGs is difficult and where our current knowledge comes from histology of post-mortem samples (Orita et al., 2002; Berger, 1993). Rodent models and, in future, other animal models, have an important role in modelling HED as they have the potential to identify key characteristics of bulla pathology, such a granulomatous inflammation induced by FB particles, which distinguishes them from a background of acute otitis media, recurrent acute otitis media and particularly chronic otitis media with effusion (glue ear), which is common in infants and young children (Bhutta et al., 2017). This understanding will better enable the evaluation of the outcomes for novel XLHED treatments in relatively small numbers of recipient patients.

## MATERIALS AND METHODS

### Mice and rat and husbandry

These studies were approved by the Roslin Institute Animal Welfare and Ethical Review Board and performed under the authority of the appropriate UK Home Office Project Licence. *Tabby* mice (*Eda^Ta/Ta^* females and *Eda^Ta/Y^* hemizygous males; collectively termed *Eda^Ta^*) were maintained as a homozygous line. FVB mice are the background inbred genetic line for the *Eda^Ta^* strain and FVB/NCrl (Charles River) mice were bred to provide control tissues. The husbandry and health surveillance of the Roslin Institute mouse colonies was as previously described (Azar et al., 2016). Timed mates were set up and the morning of plug is designated embryonic day 0.5 (E0.5). Male and female mice were used in all of the experiments.

The sparse and wavy hair (*swh*) rat strain [Kuramoto et al., 2005, 2011; WTC-*swh*/Kyo, National BioResource Project (NBRP) Rat No. 0287] was supplied by the NBRP - Rat, Kyoto University (Kyoto, Japan) and was re-derived into the Roslin Biological Resource facility by embryo transfer into Sprague Dawley rats. Specific pathogen-free colony rats were housed in individually ventilated cages (Tecniplast GR1800) under a 12 h light/12 h dark cycle, in a temperature range of 21±2°C and humidity of 55±10% with 15-20 changes of HEPA-filtered air per hour. Rats were fed irradiated Teklad 14% Protein Rodent Diet (Harlan UK Ltd) and provided with 0.2-µm-filtered water. Rats were housed on autoclaved Aspen Chip Grade 6 bedding and cage enrichment products included Aspen wood blocks, cardboard and plastic nesting materials.

Microbiological surveillance of sentinel rats followed Federation of European Laboratory Animal Science Associations (FELASA) screening guidelines and the Roslin Institute facility is free from FELASA-listed rat viruses, bacterial pathogens and parasites.

The *Edaradd* rat colony was maintained by intercrossing *Edaradd^swh/+^* rats and, out of necessity to start the colony from a small nucleus of animals, by intercrossing *Edaradd^swh/swh^* females with *Edaradd^swh/+^* males. *Edaradd^swh/swh^* females have mammary hypoplasia and wean fewer pups (Kuramoto et al., 2005). We note in our breeding programme that one *Edaradd^swh/swh^* female weaned a total of 21 pups in 5 litters of 2-8 pups. Male and female rats were used in all of the experiments.

*Edaradd* colony rats were genotyped for the mutant *swh* allele, which has a C-to-T transition in exon 6 of the rat *Edaradd* gene, causing a change of proline to serine at codon 153 of the protein (Kuramoto et al., 2011). Genotyping was performed by Transnetyx using real-time PCR with the RnEdaradd-1 Mut assay.

### Administration of agonist anti-EDAR antibody and isotype control antibodies

Two pregnant *Eda^Ta^* females were administered agonist anti-EDAR antibody (mouse IgG1 mAbEDAR1) (Kowalczyk et al., 2011) at a dose of 2 mg/kg body weight by injections on E10.5 via an intraperitoneal injection (i.p.) and at E17.5 via a tail-vein intravenous route (i.v.); *n*=2 pups were phenotyped at P22 and *n*=9 were phenotyped at P85. One pregnant *Eda^Ta^* female was administered agonist antibody (E10.5 i.p. and E17.5 i.v.) and the *n*=6 offspring pups received 2 mg antibody/kg body weight on P1 (via a subcutaneous route), P7 (i.p.) and P14 (i.p.); all six were phenotyped at P21.

Two pregnant *Eda^Ta^* females were administered isotype control Aprily-2 (mouse IgG1 anti-hAPRIL) (Kowalczyk et al., 2011) at doses of 2 mg/kg body weight by injections on E10.5 i.p. and at E17.5 i.v., and offspring (*n*=10) were phenotyped at P82-P84. Other untreated controls included P21 FVB (*n*=13), P81-P84 FVB (*n*=10), P21 *Eda^Ta^* (*n*=12) and P79-P90 *Eda^Ta^* (*n*=13) mice sampled from 2-3 litters.

### Gross examination and histological sampling

*Eda^Ta^* mice were scored for the macroscopic appearance of their hair coat and tail for the presence of hair and terminal tail kink. *Edaradd* colony rats were scored for HED phenotype as above for mice, noting that *Edaradd^swh/swh^* rats differ from EDAR signalling mutant mouse strains by having haired tails and a low penetrance of the tail kink (Kuramoto et al., 2005). P21 [from 2 litters, 3 *Edaradd^swh/swh^* (*swh/swh*), 4 *Edaradd^swh/+^* (*swh/+*)], P30 (1 litter, 5 *swh/swh*, 1 *swh/+*), P42 (from 3 litters, 4 *swh/swh*, 4 *swh/+*), P83-P85 (from 5 litters, 4 s*wh/swh*, 5 *swh/+*, 2 *+/+*) and two P226 and P250 *Edaradd^swh/swh^* females were examined.

Mouse tissues were fixed in neutral buffered formalin and bony tissues were decalcified for 2-4 days in 14% EDTA. Tissues were wax embedded for 4-µm sections and H&E staining. Haired skin from the dorsal thorax between the shoulder blades, footpad skin, eyelid and skull were sampled from P21-P22 (weaning-aged) mice and P79-P90 adult mice. Pooled tissues were multiblocked for each cohort of *Eda^Ta^* and FVB mice. Decalcified skulls were prepared for dorsal plane sections of the nasal passages, nasopharynx, auditory tubes and bullae. Nasal tissues, nasopharynx and bullae were scored for the presence (or absence) of mucosal inflammation, with or without intraluminal inflammatory exudate; the mucosa of inflamed bullae is invariably thickened in otitis media. Hyaline droplet degeneration in nasal respiratory epithelium was assessed by its presence or absence in dorsal plane sections of the nasal septum.

*Edaradd* rats were sampled for histology of the eyelid, footpad skin, dorsal thorax skin, trachea and tongue, and tissues were fixed in formalin for up to 7 days. Decalcification of P83-P85 skulls required up to 3 weeks in 14% EDTA. Tissue sections were stained with H&E or Alcian Blue-Periodic Acid Schiff (AB-PAS) stain.

### Imaging

Brightfield images were acquired using a Hamamatsu NanoZoomer slide scanner, or on an Olympus BX41 microscope equipped with a DP72 camera and Cell D software.

### Immunohistochemistry and *in situ* hybridization

Anti-mouse Bpifa1 rabbit polyclonal antibody (Musa et al., 2012) was a gift from Colin Bingle (University of Sheffield, UK). IHC was performed manually on 4-µm wax sections of P10 and P21 *Edaradd* rat skulls. Antigen retrieval (citrate buffer pH 6.0) was performed on a Histos microwave machine (100°C, 20 min); the anti-Bpifa1 rabbit primary antibody was diluted 1/800 and applied for 30 min at room temperature, and detected with an Envision Rabbit reagent kit for 40 min and Vector NovaRed chromogen.

ISH was performed on the Leica Bond Rx machine using RNAscope 2.5 LS probes and either a RED (catalogue number 322150) or BROWN (322100) detection kit according to the manufacturer's instructions (Advanced Cell Diagnostics). P5 *Eda^Ta^* skulls (fixed for 20 h and decalcified for 8 h) were examined for *Edar* (Mm-Edar 423018); P10 *Edaradd^+/+^* and P13 *Edaradd^swh/+^* tissues (fixed for 20 h, skulls decalcified for 48 h) for *Edaradd* (Rn-Edaradd 507868). Each ISH experiment was performed alongside negative *DapB* (312038) and positive *PPiB* (Mm-PPiB 313918; Rn-PPiB 313928) control probes.

We also examined auditory-tube SMGs in tissue sections of E18.5 and P1 wild-type mice (mixed C57BL/6J C3H background) generated in another study (del-Pozo et al., 2019). Duplex ISH was performed on E18.5 tissues for the non-ciliated cell marker *Bpifa1* (channel 1 probe brown) and the ciliated cell marker *Foxj1* (channel 2 probe red) with a RED/BROWN kit (322440) (del-Pozo et al., 2019). In addition, we performed separate ISH staining for *Edar* and *DapB*. To detect single (channel 1 brown) probes using the duplex RED/BROWN detection system, we reduced Amp5 time to 1 min to minimize non-specific reaction (Advanced Cell Diagnostics technical support advice). To account for the slightly higher background, we measured *Edar* and *DapB* ISH dots in consecutive serial sections of 1 auditory-tube gland primordium from ×600 jpg images. ISH dots and cells (nuclei) were counted with the ‘points’ tool to calculate the average number of dots per cell. The area (pixels) of all the ISH dots, and the area of the gland primordium, were measured with the ‘brush’ tool (Qu-Path software; https://qupath.github.io/) (Bankhead et al., 2017). These were used to calculate the percentage of the gland primordium area covered by *Edar* and *DapB* ISH signals and thereby the signal-to-noise ratio.

IHC was performed on P1 tissues for laminin, and keratins 5, 8 and 19; see del-Pozo et al. (2019) for the reagents and protocols. Histology, IHC and ISH was performed in Easter Bush Pathology laboratories that are UK NEQAS accredited.

### Morphometric analysis of rat auditory-tube SMGs

We collected rats at P21, P42 and P83-P85 to examine the development of *Edaradd^swh/swh^* and *Edaradd^swh/+^* auditory-tube SMGs using 2 or 3 animals of each genotype for each time point. Auditory-tube SMGs were serially sectioned in dorsal plane, up through the region where the auditory tubes bifurcate from the nasopharynx. The right and left auditory-tube SMGs were sampled at 40-µm steps, each SMG yielding 4-7 sections to calculate an average area per SMG. The area of the whole SMG (serous acini, mucous glands, duct epithelium, connective tissue and myoepithelial cells) (Fig. S3) was measured in slide scans of H&E-stained sections. Step sections separated by 40 µm represent separate SMG cell populations. As the area data was normally distributed, we calculated an average per slide for each SMG. AB-PAS sections were used to measure area occupied by AB-positive mucous and PAS-positive serous cells. For stain analysis, images were separated into different channels using a colour deconvolution method described in Ruifrok and Johnston (2001). Briefly, the RGB colour vectors corresponding to each stain (AB, PAS and background) were determined using regions of interest in a subset of the sample. The RGB values obtained for each colour were then recorded in a macro that was used to analyse the complete data set by producing the following values: total gland tissue surface, total AB-stained surface and total PAS-stained surface.

Slide scans were used to measure nasopharynx width immediately rostral to the opening or the auditory tubes as the distance between the lateral walls formed by the basisphenoid bones (Bab et al., 2007) using Qu-Path (Bankhead et al., 2017) and ImageJ. The P30 cohort had only 1 *Edaradd^swh/+^* and 5 *Edaradd^swh/swh^* rats and was excluded from the morphometric analysis.

### Statistical analysis

The statistical tests were chosen after performing D'Agostino and Pearson omnibus normality tests. Group sizes for rat SMG area data were too small to test for normality so we used Mann–Whitney tests for their analysis. The summary statistics and the statistical tests used for each data set are described in the figure legends. Fisher's exact tests were used to compare disease frequencies. Two-tailed tests were used throughout and test values of *P*<0.05 were considered to be statistically significant. Graphs and statistics were generated using GraphPad Prism and Vassar Stats (http://www.vassarstats.net/).

## Supplementary Material

Supplementary information

## References

[DMM037804C1] Azar A., Piccinelli C., Brown H., Headon D. and Cheeseman M. (2016). Ectodysplasin signalling deficiency in mouse models of Hypohidrotic Ectodermal Dysplasia leads to middle ear and nasal pathology. *Hum. Mol. Genet.* 25, 3564-3577. 10.1093/hmg/ddw202 27378689 PMC5179950

[DMM037804C2] Bab I., Hajbi-Yonissi C., Gabet Y. and Müller R. (2007). *Micro-Tomographic Atlas of the Mouse Skeleton*. New York, USA: Springer.

[DMM037804C3] Bankhead P., Loughrey M. B., Fernández J. A., Dombrowski Y., McArt D. G., Dunne P. D., McQuaid S., Gray R. T., Murray L. J., Coleman H. G. et al. (2017). QuPath: open source software for digital pathology image analysis. *Sci. Rep.* 7, 16878 10.1038/s41598-017-17204-5 29203879 PMC5715110

[DMM037804C4] Bartlett J. A., Meyerholz D. K., Wohlford-Lenane C. L., Naumann P. W., Salzman N. H. and McCray P. B. Jr. (2015). Increased susceptibility to otitis media in a Splunc1-deficient mouse model. *Dis. Model. Mech.* 8, 501-508. 10.1242/dmm.019646 25765466 PMC4415896

[DMM037804C5] Berger G. (1993). Eustachian tube submucosal glands in normal and pathological temporal bones. *J. Laryngol. Otol.* 107, 1099-1105. 10.1017/S002221510012540X 8288996

[DMM037804C6] Bhutta M. F., Thornton R. B., Kirkham L.-A. S., Kerschner J. E. and Cheeseman M. T. (2017). Understanding the aetiology and resolution of chronic otitis media from animal and human studies. *Dis. Model. Mech.* 10, 289-1300. 10.1242/dmm.029983 PMC5719252 29125825

[DMM037804C7] Callea M., Teggi R., Yavuz I., Tadini G., Priolo M., Crovella S., Clarich G. and Grasso D. L. (2013). Ear nose throat manifestations in hypoidrotic ectodermal dysplasia. *Int. J. Pediatr. Otorhinolaryngol.* 77, 1801-1804. 10.1016/j.ijporl.2013.09.004 24080322

[DMM037804C8] Chamanza R. and Wright J. A. (2015). A review of the comparative anatomy, histology, physiology and pathology of the nasal cavity of rats, mice, dogs and non-human primates. Relevance to inhalation toxicology and human health risk assessment. *J. Comp. Pathol.* 153, 287-314. 10.1016/j.jcpa.2015.08.009 26460093

[DMM037804C9] Chang S. H., Jobling S., Brennan K. and Headon D. J. (2009). Enhanced Edar signalling has pleiotropic effects on craniofacial and cutaneous glands. *PLoS. ONE* 4, e7591 10.1371/journal.pone.0007591 19855838 PMC2762540

[DMM037804C10] del-Pozo J., MacIntyre N., Azar A., Glover J., Milne E. and Cheeseman M. (2019). Chronic otitis media is initiated by a bulla cavitation defect in the FBXO11 mouse model. *Dis. Model. Mech.* 12, dmm038315 10.1242/dmm.038315 30898767 PMC6451434

[DMM037804C11] Grüneberg H. (1971). The glandular aspects of the tabby syndrome in the mouse. *J. Embryol. Exp. Morphol.* 25, 1-19. 5548211

[DMM037804C12] Hadji Rasouliha S., Bauer A., Dettwiler M., Welle M. M. and Leeb T. (2018). A frameshift variant in the EDA gene in Dachshunds with X-linked hypohidrotic ectodermal dysplasia. *Anim. Genet.* 49, 651-654 10.1111/age.12729 30276836

[DMM037804C13] Henningsen E., Svendsen M. T., Lildballe D. L. and Jensen P. K. (2014). A novel mutation in the EDAR gene causes severe autosomal recessive hypohidrotic ectodermal dysplasia. *Am. J. Med. Genet. A.* 164A, 2059-2061. 10.1002/ajmg.a.36582 24764207

[DMM037804C14] Jaskoll T., Zhou Y.-M., Trump G. and Melnick M. (2003). Ectodysplasin receptor-mediated signaling is essential for embryonic submandibular salivary gland development. *Anat. Rec. A Discov. Mol. Cell Evol. Biol.* 271, 322-331. 10.1002/ar.a.10045 12629675

[DMM037804C15] Karlskov-Mortensen P., Cirera S., Nielsen O. L., Arnbjerg J., Reibel J., Fredholm M. and Agerholm J. S. (2011). Exonization of a LINE1 fragment implicated in X-linked hypohidrotic ectodermal dysplasia in cattle. *Anim. Genet.* 42, 578-584. 10.1111/j.1365-2052.2011.02192.x 22034998

[DMM037804C16] Kowalczyk C., Dunkel N., Willen L., Casal M. L., Mauldin E. A., Gaide O., Tardivel A., Badic G., Etter A.-L., Favre M. et al. (2011). Molecular and therapeutic characterization of anti-ectodysplasin A receptor (EDAR) agonist monoclonal antibodies. *J. Biol. Chem.* 286, 30769-30779. 10.1074/jbc.M111.267997 21730053 PMC3162438

[DMM037804C17] Kowalczyk-Quintas C. and Schneider P. (2014). Ectodysplasin A (EDA)-EDA receptor signalling and its pharmacological modulation. *Cytokine Growth Factor. Rev.* 25, 195-203. 10.1016/j.cytogfr.2014.01.004 24508088

[DMM037804C18] Kowalczyk-Quintas C., Willen L., Dang A. T., Sarrasin H., Tardivel A., Hermes K., Schneider H., Gaide O., Donzé O., Headon D. J. et al. (2014). Generation and characterization of function blocking anti-ectodysplasin A (EDA) monoclonal antibodies that induce ectodermal dysplasia. *J. Biol. Chem.* 289, 4273-4285. 10.1074/jbc.M113.535740 24391090 PMC3924290

[DMM037804C19] Kuramoto T., Morimura K., Nomoto T., Namiki C., Hamada S., Fukushima S., Sugimura T., Serikawa T. and Ushijima T. (2005). Sparse and wavy hair: a new model for hypoplasia of hair follicle and mammary glands on rat chromosome 17. *J. Hered.* 96, 339-345. 10.1093/jhered/esi053 15829729

[DMM037804C20] Kuramoto T., Yokoe M., Hashimoto R., Hiai H. and Serikawa T. (2011). A rat model of hypohidrotic ectodermal dysplasia carries a missense mutation in the Edaradd gene. *BMC Genet.* 12, 91 10.1186/1471-2156-12-91 22013926 PMC3224228

[DMM037804C21] Li J.-D., Hermansson A., Ryan A. F., Bakaletz L. O., Brown S. D., Cheeseman M. T., Juhn S. K., Jung T. T. K., Lim D. J., Lim J. H. et al. (2013). Panel 4: recent advances in otitis media in molecular biology, biochemistry, genetics, and animal models. *Otolaryngol. Head Neck Surg.* 148 Suppl. 4, E52-E63. 10.1177/0194599813479772 23536532 PMC4006668

[DMM037804C22] Majumder K., Shawlot W., Schuster G., Harrison W., Elder F. F. and Overbeek P. A. (1998). YAC rescue of downless locus mutations in mice. *Mamm. Genome* 9, 863-868. 10.1007/s003359900884 9799834

[DMM037804C23] Martini A., Magnan G. and Peserico A. (1984). Ozena as presenting symptom of a rare and severe genetic disease: hypohidrotic ectodermal dysplasia. *Int. J. Pediatr. Otorhinolaryngol.* 8, 97-103. 10.1016/S0165-5876(84)80030-0 6500829

[DMM037804C24] Mehta U., Brunworth J., Fete T. J. and Sindwani R. (2007a). Head and neck manifestations and quality of life of patients with ectodermal dysplasia. *Otolaryngol. Head Neck Surg.* 136, 843-847. 10.1016/j.otohns.2006.11.038 17478227

[DMM037804C25] Mehta U., Brunworth J., Lewis R. A. and Sindwani R. (2007b). Rhinologic manifestations of ectodermal dysplasia. *Am. J. Rhinol.* 21, 55-58. 10.2500/ajr.2007.21.2989 17283562

[DMM037804C26] Mou C., Thomason H. A., Willan P. M., Clowes C., Harris W. E., Drew C. F., Dixon J., Dixon M. J. and Headon D. J. (2008). Enhanced ectodysplasin-A receptor (EDAR) signaling alters multiple fiber characteristics to produce the East Asian hair form. *Hum. Mutat.* 29, 1405-1411. 10.1002/humu.20795 18561327

[DMM037804C27] Musa M., Wilson K., Sun L., Mulay A., Bingle L., Marriott H. M., LeClair E. E. and Bingle C. D. (2012). Differential localisation of BPIFA1 (SPLUNC1) and BPIFB1 (LPLUNC1) in the nasal and oral cavities of mice. *Cell Tissue Res.* 350, 455-464. 10.1007/s00441-012-1490-9 22986921 PMC3505551

[DMM037804C28] Orita Y., Sando I., Hirsch B. E., Miura M., Hasebe S. and Balaban C. D. (2002). Postnatal development of the eustachian tube glands. *Laryngoscope* 112, 1647-1652. 10.1097/00005537-200209000-00022 12352680

[DMM037804C29] Park K. and Lim D. J. (1992). Development of the mucociliary system in the eustachian tube and middle ear: murine model. *Yonsei Med. J.* 33, 64-71. 10.3349/ymj.1992.33.1.64 1502832

[DMM037804C30] Ruifrok A. C. and Johnston D. A. (2001). Quantification of histochemical staining by color deconvolution. *Anal. Quant. Cytol. Histol.* 23, 291-299. 11531144

[DMM037804C31] Schneider H., Faschingbauer F., Schuepbach-Mallepell S., Körber I., Wohlfart S., Dick A., Wahlbuhl M., Kowalczyk-Quintas C., Vigolo M., Kirby N. et al. (2018). Prenatal correction of X-linked hypohidrotic ectodermal dysplasia. *N. Engl. J. Med.* 378, 1604-1610. 10.1056/NEJMoa1714322 29694819

[DMM037804C32] Seeliger F., Drögemüller C., Tegtmeier P., Baumgärtner W., Distl O. and Leeb T. (2005). Ectodysplasin-1 deficiency in a German Holstein bull associated with loss of respiratory mucous glands and chronic rhinotracheitis. *J. Comp. Pathol.* 132, 346-349. 10.1016/j.jcpa.2004.11.001 15893993

[DMM037804C33] Shin J. J. and Hartnick C. J. (2004). Otologic manifestations of ectodermal dysplasia. *Arch, Otolaryngol, Head Neck Surg.* 130, 1104-1107. 10.1001/archotol.130.9.1104 15381599

[DMM037804C34] St. Clair M. B. G. and Morgan K. T. (1992). Changes in the upper respiratory tract. In *Pathobiology of the Aging Rat*, Vol. 1 (ed. Mohr U., Dungworth C. C. and Capen C. C.), pp. 111-127. Washington, DC, USA: ILSI Press.

[DMM037804C35] Swee L. K., Ingold-Salamin K., Tardivel A., Willen L., Gaide O., Favre M., Demotz S., Mikkola M. and Schneider P. (2009). Biological activity of ectodysplasin A is conditioned by its collagen and heparan sulfate proteoglycan-binding domains. *J. Biol. Chem.* 284, 27567-27576. 10.1074/jbc.M109.042259 19657145 PMC2785685

[DMM037804C36] Waluk D. P., Zur G., Kaufmann R., Welle M. M., Jagannathan V., Drögemüller C., Müller E. J., Leeb T. and Galichet A. (2016). A splice defect in the EDA gene in dogs with an X-linked hypohidrotic ectodermal dysplasia (XLHED) phenotype. *G3 (Bethesda)* 6, 2949-2954. 10.1534/g3.116.033225 27449516 PMC5015951

