# Supplementary material for: Role of ectodysplasin signalling in middle ear and nasal pathology in rat and mouse models of hypohidrotic ectodermal dysplasia
Source: Dis Model Mech. 2019 Apr 25;12(4):dmm037804. doi: 10.1242/dmm.037804 (PMC6505480; doi:10.1242/dmm.037804)
Supplement: Supplementary information [file dmm-12-037804-s1.pdf]

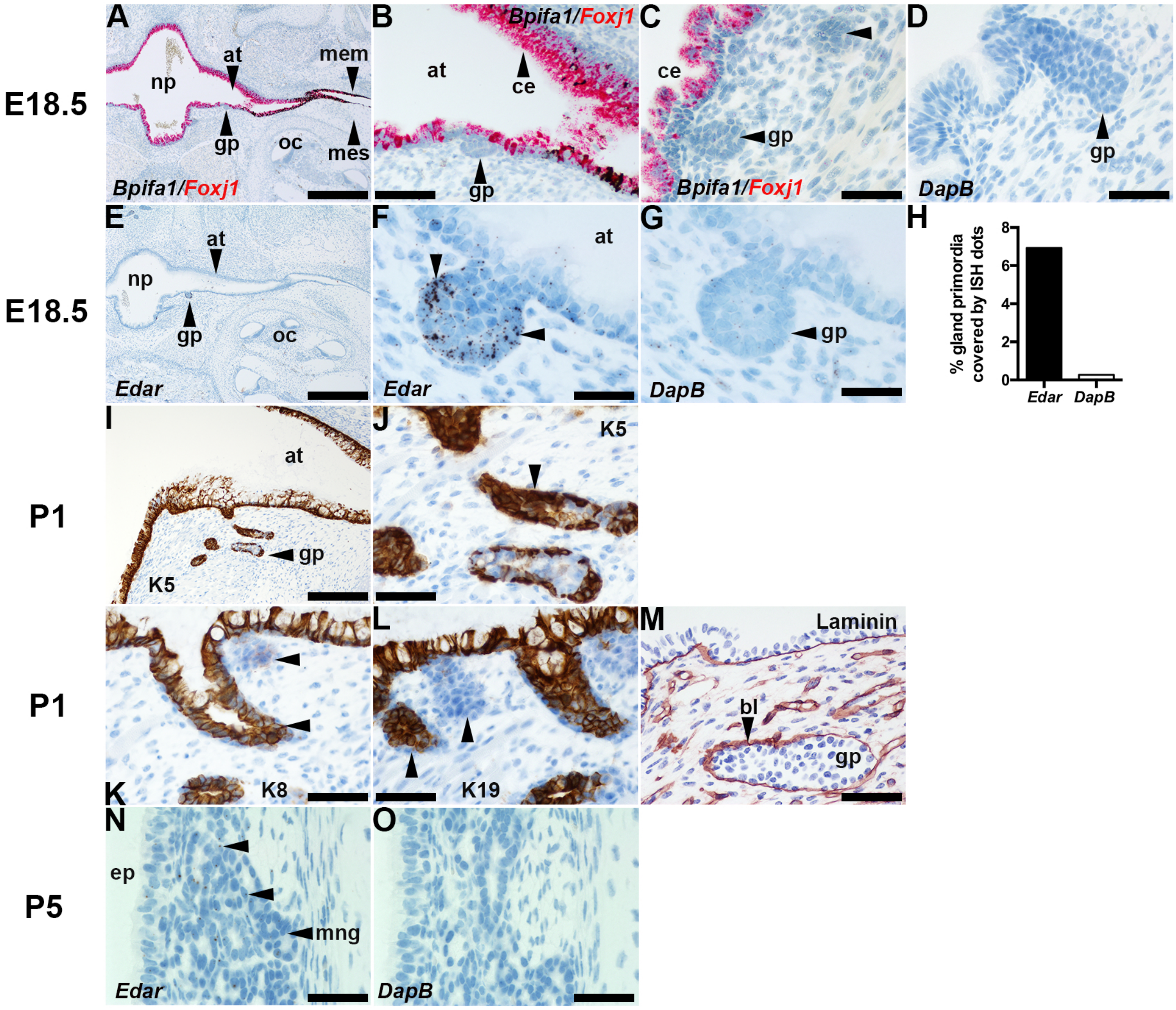

Fig. S1. *Edar* expression in E18.5 gland primordia and P5 nasal tissues.
(A-H) E18.5 wild-type mouse (mixed C57BL/6J C3H background) images from four heads. (A-G) Auditory-tube SMG primordia appear as buds of cells that extend into submucosal connective tissue from the ciliated epithelium of the auditory-tube (A,B,E-G) and nasopharynx (C,D) (arrowheads). (A-C) *Foxj1* and *Bpifa1* duplex ISH; *Foxj1* is a marker for ciliated cells and *Bpifa1* is a non-ciliated epithelial cell marker. (D) Single plex ISH negative control *DapB*. (E,F) *Edar* ISH signals in a representative gland primordium appear as small dots or dot clusters (arrowheads in F), (G) *DapB*. Note F and G are serial sections of the same gland primordium and there are ~12 times as many *Edar* ISH dots per cell (F) than background *DapB* (G) (2.7 versus 0.23 per cell in n=57 and n=56 cells respectively). (H) *Edar* ISH signals (dots and dot clusters) cover a ~25 times greater area than *DapB* dots (6.9% versus 0.27% coverage of gland primordium area). (I-M) P1 wild-type mouse auditory-tube SMG gland primordia. (I,J) Keratin 5 IHC, (I,J) low and high power images of the same tissue section.
(K) Keratin 8 and (L) Keratin 19 appear to stain some gland primordia but not others (arrowheads).
(M) Gland primordia have a basal lamina that stains for laminin by IHC.
(N,O) P5 *EdaTa* nasal tissues (N) *Edar* (ISH dots indicated by unlabelled arrowheads) and (O) negative control *DapB* ISH.
Abbreviations: at, auditory-tube; bl, basal lamina; ce, ciliated epithelium; ep, nasal epithelium; gp, gland primordia; mem, middle ear mucosa; mes, middle ear mesenchyme; mng, medial nasal gland; np, nasopharynx; oc, otic capsule.
Scale bars: (A,E) 500 µm; (I) 200 µm; (B) 100 µm; (C,D,J-M) 50 µm; (F,G,N,O) 20 µm.

Disease Models & Mechanisms • Supplementary information

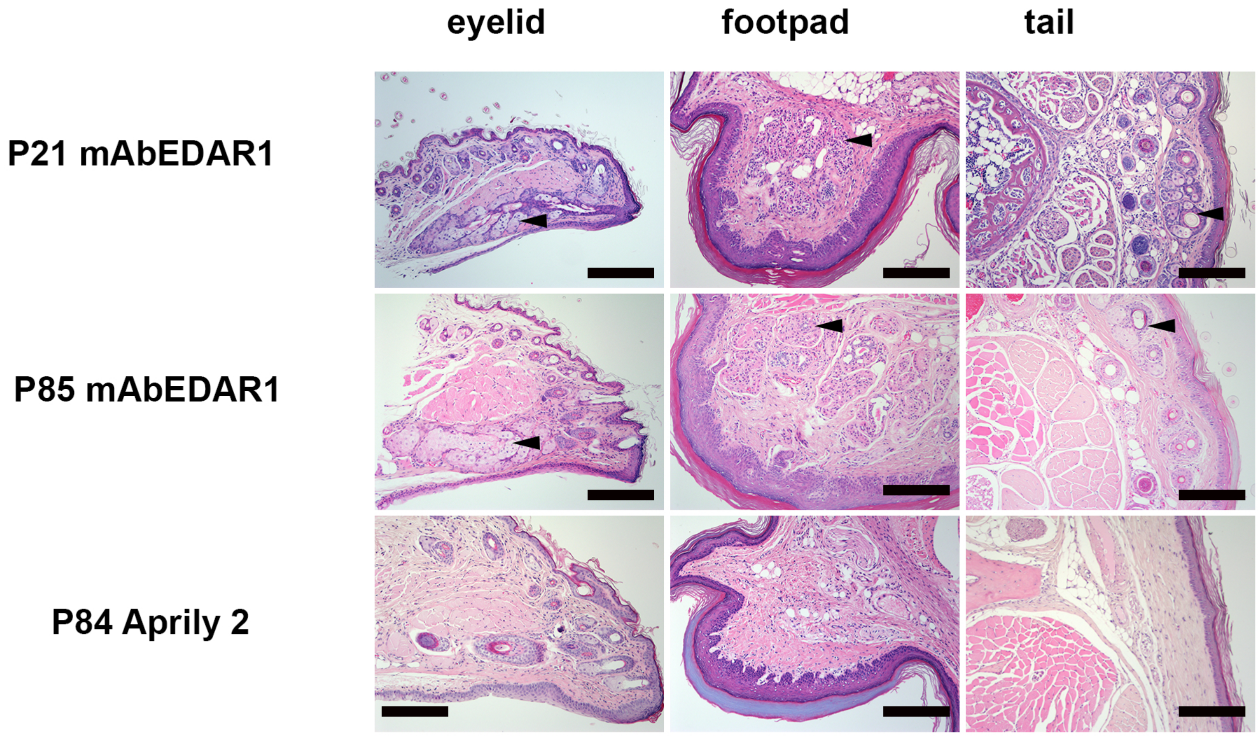

Fig. S2. EDAR signalling agonist antibodies rescue cutaneous phenotypes in *Eda Ta* mice
Eyelid Meibomian glands (arrowheads), footpad eccrine sweat glands (arrowheads) and tail hair and sebaceous glands (arrowheads) are rescued in *Eda Ta* mice treated with agonist anti-EDAR antibody (mAbEDAR1), but not when treated with isotype control Aprily 2 antibody.
Scale bars: 500 µm.

Disease Models & Mechanisms • Supplementary information

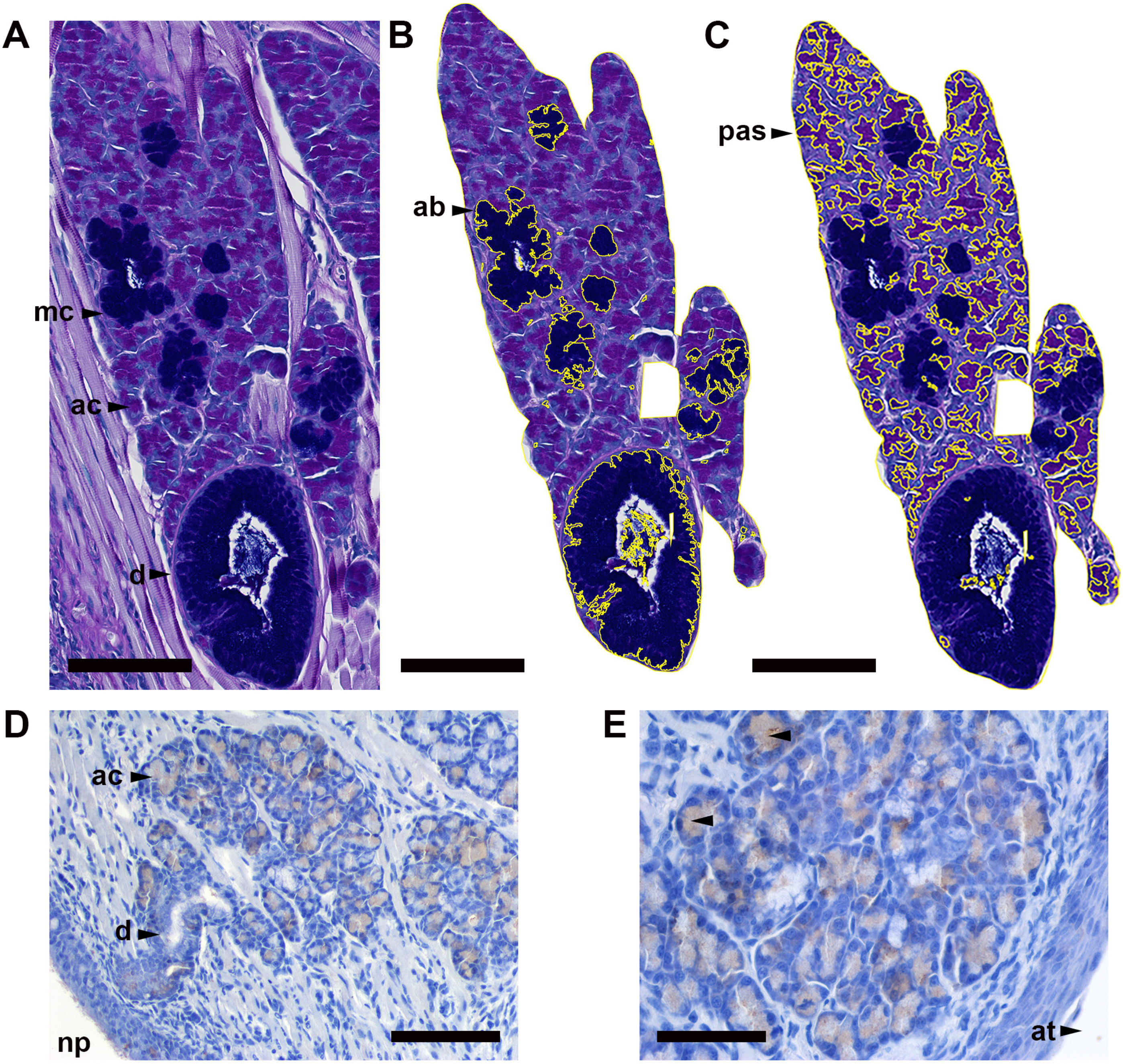

Fig. S3. *Edaradd* rat auditory-tube SMGs
(A) AB-PAS stained P85 *Edaradd swh/swh* auditory-tube SMG with mucous cells, mucous duct and serous acini.
(B) Image J analysis which delineates the SMG region of interest and segments areas of AB-positive mucus and (C) PAS-positive acini with yellow lines. (D,E) Bpifa1 IHC staining of P21 *Edaradd swh/swh* auditory-tube SMG acinar cells, E higher magnification shows staining of apical cytoplasm that contains secretory granules (unlabelled arrowheads).
Abbreviations: ab, AB-positive mucus; ac, acini; at, auditory tube; d, duct; mc, mucous cells; np, nasopharynx; pas, PAS-positive acinar secretory granules.
Scale bars: (A,B,C,E) 100 µm; (D) 50 µm.

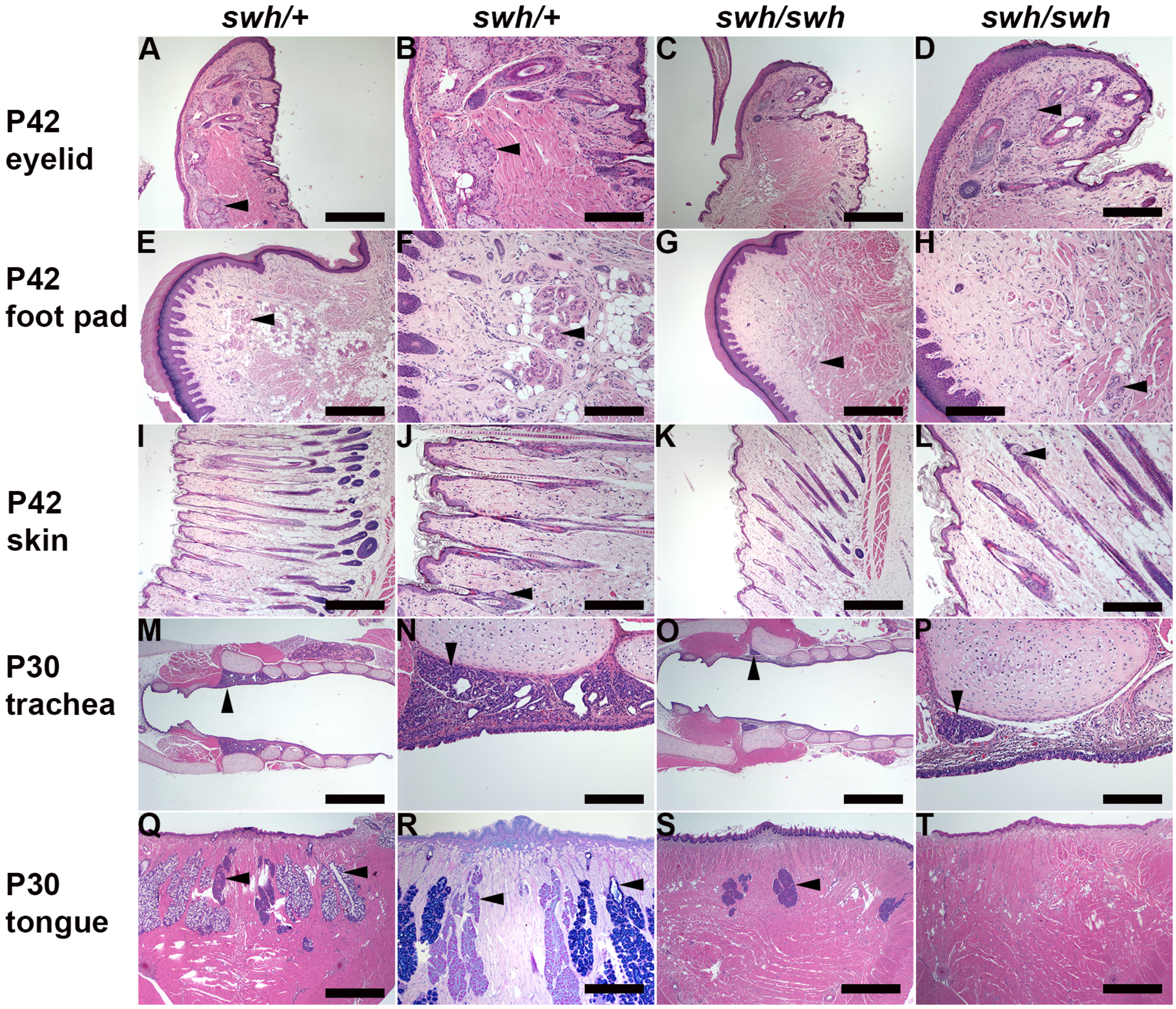

Fig. S4. HED phenotypes in Edaradd swh/swh rats
(A,B) Meibomian glands (arrowheads) found in *swh/+* eyelid are inapparent in (C,D) *swh/swh* eyelid; in panel D arrowhead indicates sebaceous gland adjacent to hair follicle. (E,F) Eccrine sweat glands (arrowheads) are more numerous in *swh/+* footpad than those in (G,H) *swh/swh* footpad. (I,J) Hair follicles are more numerous *swh/+* skin than those in (K,L) *swh/swh* skin; both genotypes have sebaceous glands (arrowheads in J and L). (M,N) Tracheal SMGs (arrowheads) are larger in *swh/+* than those in (O,P) *swh/swh* rats. (Q,R) Tongue SMGs (arrowheads) in *swh/+* are larger and have AB-PAS-positive serous and mucous cell populations (R), whereas SMGs in *swh/swh* tongue (S) are hypoplastic and lack mucous glands (arrowhead indicates serous gland), or are absent (T).
Note panels (A,B), (C,D), (E,F), (G,H), (I,J), (K,L), (M,N) and (O,P) are low and high magnification image pairs.
Scale Bars: (M,O,Q,S,T) 1000 µm; (A,C,E,G,I,K,R) 500 µm; (B,D,F,H,J,L,N,P) 200 µm.

Disease Models & Mechanisms • Supplementary information
